# Pacemaker implantation after cardiac surgery: a contemporary, nationwide perspective

**DOI:** 10.1136/heartjnl-2024-325321

**Published:** 2025-03-29

**Authors:** Amar Taha, Alice David, Sigurdur Ragnarsson, Piotr Szamlewski, Shabbar Jamaly, Jan Gustav Smith, Susanne J Nielsen, Anders Jeppsson, Andreas Martinsson

**Affiliations:** 1 Department of Molecular and Clinical Medicine, Sahlgrenska Academy, Gothenburg, Sweden; 2 Department of Cardiology, Sahlgrenska University Hospital, Gothenburg, Sweden; 3 Division of Cardiothoracic Surgery, Skåne University Hospital Lund, Lund, Sweden; 4 Department for Clinical Sciences, Lund University, Lund, Sweden; 5 Department of Cardiology, Skåne University Hospital Lund, Lund, Sweden; 6 Department of Cardiothoracic Surgery, Sahlgrenska University Hospital, Gothenburg, Sweden

**Keywords:** Cardiac surgery, Arrhythmias, Cardiac, Bradycardia

## Abstract

**Background:**

Cardiac surgery carries a heightened risk of bradyarrhythmias, but current permanent pacemaker (PPM) implantation estimates rely on non-contemporary studies. This study primarily aimed to explore the incidence and indications for PPM implantation at 30 days and 1 year after different cardiac surgical procedures in a modern cohort. Secondary outcomes were PPM incidence at 10 years and time from cardiac surgery to PPM implantation.

**Methods:**

This nationwide population-based study included all patients in Sweden who from 2006 to 2020 underwent first-time coronary artery bypass grafting (CABG) and/or valvular surgery. Patients with previous PPM, previous or later implantable cardioverter-defibrillator (ICD) and those who underwent heart transplantation were excluded.

**Results:**

Overall, 76 447 patients were included, out of which 8.2% (n=6271) received a PPM. The cumulative incidence of PPM implantation was 2.9%, 3.8% and 9.5% at 30 days, 1 year and 10 years following cardiac surgery, respectively. The main PPM indication was atrioventricular block. Tricuspid valve surgery exhibited the highest cumulative incidence for PPM both at 30 days (6.8%, 95% CI 4.3% to 10.0%) and 1 year (8.8%, 95% CI 6.0% to 12.0%) surpassing mitral valve surgery (30 day 5.3%, 95% CI 4.7% to 6.0%; 1 year 6.5%, 95% CI 5.8% to 7.3%), aortic valve surgery (30 day 4.8%, 95% CI 4.5% to 5.1%; 1 year 6.0%, 95% CI 5.6% to 6.3%) and CABG (30 day 0.74%, 95% CI 0.6% to 0.8%; 1 year 1.3%, 95% CI 1.2% to 1.3%). The incidence following combined operations (multiple valves and/or CABG) was 6.5% (95% CI 6.0% to 6.9%) and 8.1% (95% CI 7.7% to 8.6%) at 30 days and 1 year, respectively. Concomitant ablation surgery increased the risk even further (adjusted HR 9.2, 95% CI 7.9 to 10.6; p<0.001).

**Conclusions:**

The need for PPM after cardiac surgery is substantial, primarily due to atrioventricular block. Tricuspid valve surgery is associated with the highest risk for PPM among isolated procedures. Combined procedures and concomitant surgical ablation further increase that risk.

**WHAT IS ALREADY KNOWN ON THIS TOPIC**

- Currently known permanent pacemaker implantation estimates after cardiac surgery are widely varying and based on outdated data.

**WHAT THIS STUDY ADDS**

- Permanent pacemaker implantation after cardiac surgery is not uncommon, primarily due to atrioventricular block. Tricuspid valve surgery showed the highest cumulative incidence at both 30 days and 1 year, surpassing other isolated procedures. The risk was further elevated with combined cardiac surgeries and concurrent ablation procedures.

**HOW THIS STUDY MIGHT AFFECT RESEARCH, PRACTICE OR POLICY**

- The findings from this nationwide, contemporary study offer valuable insights for informing patients awaiting cardiac surgery about potential postoperative complications. Identifying high-risk patients allows for targeted postoperative care, including closer monitoring for conduction disturbances.

## Introduction

 Cardiac surgery is associated with a risk of non-transient bradycardia requiring implantation of a permanent pacemaker (PPM).^1^ Due to the proximity of the heart valves to the cardiac conduction system, surgical trauma during valvular surgery may lead to atrioventricular block.^2^ Also, injury to the sinoatrial node may occur during lateral atriotomy or transseptal approaches targeting the mitral valve.^1^ Alternatively, an underlying pathology could be detected by extensive monitoring after cardiac surgery, revealing a previously undiagnosed conduction disorder.

Current PPM implantation estimates following cardiac surgery are based on studies with data from the late 1990s and early 2000s, with considerable variations in the reported rates.^3^,^5^ Recent analyses have also yielded inconsistent results, often based on earlier data, single-centre studies or specific procedures.^16^,^8^ These discrepancies stem from differences in study populations (valvular vs non-valvular surgeries, in-hospital vs post-discharge PPM implantation), follow-up durations, study endpoints and the inclusion of patients with implantable cardioverter-defibrillators (ICDs).^7^ A registry-based study from 2017 compared the risk of PPM following valvular surgery versus coronary artery bypass grafting (CABG) over 10 years.^9^ However, it excluded patients undergoing mitral valve surgery or tricuspid annuloplasty and did not report PPM indications. In cardiac surgery patients, PPM implantation may also be linked to worse outcomes, as prior research found that PPM increased the risk of all-cause mortality and heart failure hospitalisation after aortic valve surgery.^6^

This study aimed to explore the PPM implantation rates and indications at 30 days, 1 year, and beyond the first postoperative year following a wide range of cardiac surgical procedures in a large contemporary nationwide cohort. The risk in cardiac surgery patients was compared with an age-matched and sex-matched cohort from the general population to investigate the long-term risk for PPM associated with cardiac surgery.

## Methods

### Study design and data sources

This was an observational, nationwide study using prospectively collected data from four mandatory Swedish registries: the Swedish Cardiac Surgery Registry, the National Patient Registry, the Swedish ICD & Pacemaker Registry and the Swedish Total Population Registry.

The Swedish Cardiac Surgery Registry is part of the Swedish Web-system for Enhancement and Development of Evidence-based care in Heart disease Evaluated According to Recommended Therapies (SWEDEHEART) registry. It has, since 1992, contained detailed demographic and procedural information for all cardiac operations in Sweden with high validity and >99% coverage^10 11^ and was used, in this study, to identify patients and characterise the type of surgery performed.

Preprocedural comorbidities were collected from the National Patient Registry which contains all hospital-based diagnoses registered using the International Classification of Diseases, Tenth Revision since 1987 for inpatient and 2001 for outpatient settings. Reporting to this registry is mandatory and with complete national coverage.^12^

Information on the type of cardiac arrhythmia devices, implantation date and indication was obtained from the Swedish ICD & Pacemaker Registry, which since 2002, has had an online platform (http://www.pacemakerregistret.se). All implanting centres in Sweden report to the registry and the data in this registry are regularly monitored.^13^ Finally, the Swedish Total Population Registry^14^ was used to extract the control population and mortality data. Individual patient data from the registries were linked using the unique personal identification number that all residents in Sweden possess.

This manuscript was composed following the recommendations in the Strengthening the Reporting of Observational Studies in Epidemiology (STROBE) statement.^15^

### Study cohort

**Figure 1:**
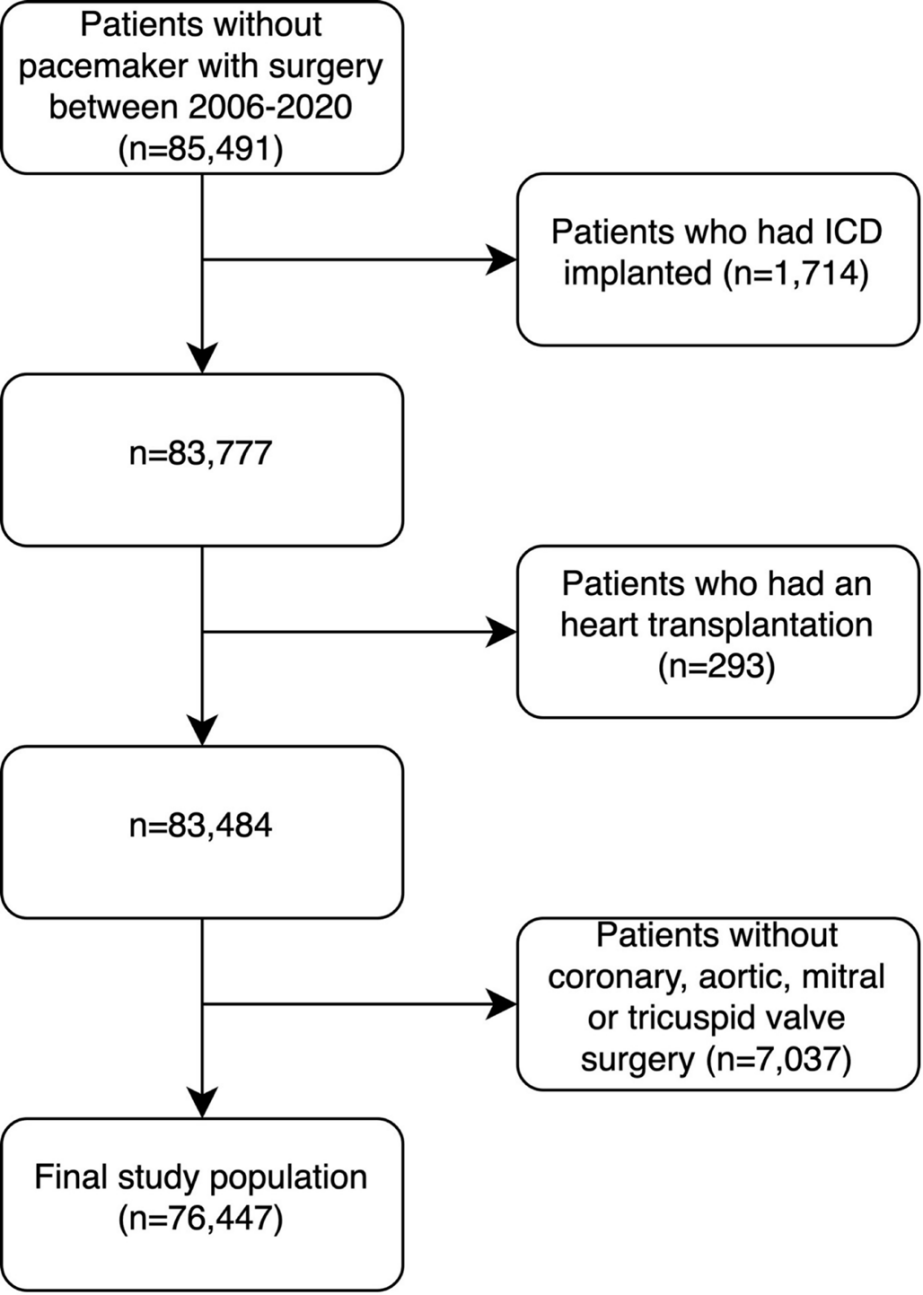
Flow chart of included and excluded patients. ICD, implantable cardioverter-defibrillator.

All adult patients in Sweden, without a previously implanted PPM or ICD, and who underwent first-time cardiac surgery between January 2006 and December 2020 were identified. The following groups of patients were excluded: those who underwent (1) heart transplantation, (2) any cardiac surgery procedure that did not include stand-alone or combination of CABG, mitral valve surgery, aortic valve surgery or tricuspid valve surgery and (3) subsequent ICD implantation. The final population comprised patients undergoing CABG, mitral, aortic or tricuspid valve surgery or a combination thereof. A flow chart of included and excluded patients is presented in figure 1.

To assess long-term PPM risk beyond the first year, age-matched and sex-matched controls with no prior cardiac surgery were compared with surgical patients, with inclusion dates aligned to surgery dates. Both groups had to survive the first postoperative year with at least 1 year of follow-up, excluding those with a previous pacemaker or one implanted within a year postsurgery.

### Outcome measures

The primary study outcomes were the cumulative incidence of pacemaker implantation at 30 days and 1 year. Secondary outcomes were pacemaker implantation at long-term follow-up and time from the cardiac surgical procedure to PPM implantation. Additionally, the PPM indication was explored. Using the control population, freedom from PPM after the first postoperative year was compared between patients and control subjects.

### Statistical analysis

For baseline characteristics, continuous variables were either presented as means with SD or medians with IQR, depending on the distribution, and categorical variables as numbers and percentages. The distribution of the variables was evaluated graphically using histograms and Q–Q plots due to the large database. Point estimates for incidence rates were calculated by dividing the number of events by follow-up years. The corresponding 95% CIs were calculated under the assumption of a Poisson distribution. To allow for competing risks, cumulative incidence was used to assess incidence over time at 30 days, 1 year and 10 years. Two variables had missing data, body mass index (7.3%) and left ventricular ejection fraction (0.6%). Missing data were handled by multiple imputation, using the chained equation method, and employing either polytomous regression or logistic regression depending on the data. Competing risks regression was performed using the Fine-Gray proportional subdistribution hazards model in which all-cause mortality was considered the competing risk. The analysis was stratified on type of surgery and adjusted for age and sex. The analysis evaluated if the risk of pacemaker implantation associated with different types of surgery increased or decreased over the study period. Cox proportional hazard models were used to calculate age-adjusted and sex-adjusted hazard ratios (aHRs) with 95% CI when comparing cardiac surgery patients with control subjects. Since the aim of the Cox regression analysis was to evaluate the long-term difference, the baseline was set at 1 year after surgery. This analysis had no missing data in that analysis. Logistic regression analysis adjusted for age and sex was used to establish an association between the year of operation and incident PPM.

Patients were classified into six groups based on the type of cardiac surgical procedure: (1) isolated CABG, (2) isolated aortic valve surgery, (3) isolated mitral valve surgery, (4) isolated tricuspid valve surgery, (5) combined valve surgery or combined valve surgery and CABG and (6) concomitant arrhythmia surgery (independently of the primary procedure). Since arrhythmia surgery was almost exclusively combined with other procedures, a Cox regression analysis was performed to evaluate the impact that arrhythmia surgery had on each of the other surgery groups.

All tests were two-tailed and interpreted at the 0.05 significance level. All analyses were performed using R V.4.2.3 (R Foundation for Statistical Computing, Vienna, Austria).^16^

### Patient and public involvement

Patients and the public were not involved in the design, conduct, reporting or dissemination plans of this study.

### Data sharing statement

The used data will be made available on reasonable request, subject to approval by the SWEDEHEART Registry, the Swedish ICD & Pacemaker Registry, the Swedish National Board of Health and Welfare and the Ethical Review Authority.

## Results

### Cohort description

In total, 76 447 patients were included. Median follow-up was 5.8 years (IQR 2.6–9.7). The mean age (SD) was 68 (11), and 25.5% were females. Isolated CABG was performed in 41 946 (54.9%) patients, isolated mitral valve surgery in 4695 (6.1%), isolated aortic valve surgery in 17 243 (22.6%) and isolated tricuspid valve surgery in 309 (0.4%). Combined valve or valve and coronary surgery were performed in 9867 (12.9%) patients, and 2387 (3.1%) underwent concomitant surgical ablation (table 1). A PPM was implanted in 8.2% (n=6271) of the patients during the entire follow-up period.

**Table 1:** Baseline characteristics

	No pacemaker (n=73 544)	Pacemaker within 1 year (n=2903)	Overall (n=76 447)
Sex			
Female	18 603 (25.3)	886 (30.5)	19 489 (25.5)
Male	54 941 (74.7)	2017 (69.5)	56 958 (74.5)
Age (years)			
Mean (SD)	68 (11)	70 (11)	68 (11)
Body mass index (kg/m^2^)			
Underweight (<20)	1959 (2.7)	122 (4.2)	2081 (2.7)
Normal (20–25)	22 136 (30.1)	1015 (35.0)	23 151 (30.3)
Overweight (25–30)	32 382 (44.0)	1183 (40.8)	33 565 (43.9)
Obese (>30)	17 067 (23.2)	583 (20.1)	17 650 (23.1)
LVEF			
>50%	52 893 (71.9)	1959 (67.5)	54 852 (71.8)
<50%	20 651 (28.1)	944 (32.5)	21 595 (28.2)
Heart failure	15 921 (21.6)	1021 (35.2)	16 942 (22.2)
Atrial fibrillation	26 808 (36.5)	1660 (57.2)	28 468 (37.2)
Prior stroke	6495 (8.8)	312 (10.7)	6807 (8.9)
Prior myocardial infarction	26 344 (35.8)	634 (21.8)	26 978 (35.3)
Chronic respiratory failure	8210 (11.2)	364 (12.5)	8574 (11.2)
Renal failure	4854 (6.6)	308 (10.6)	5162 (6.8)
Diabetes mellitus	18 119 (24.6)	605 (20.8)	18 724 (24.5)
Hypertension	47 848 (65.1)	1836 (63.2)	49 684 (65.0)
Type of surgery			
Isolated CABG	41 392 (56.3)	554 (19.1)	41 946 (54.9)
Isolated aortic valve surgery	16 218 (22.1)	1025 (35.3)	17 243 (22.6)
Isolated mitral valve surgery	4391 (6.0)	304 (10.5)	4695 (6.1)
Isolated tricuspid valve surgery	282 (0.4)	27 (0.9)	309 (0.4)
Combined cardiac surgery	9145 (12.4)	722 (24.9)	9867 (12.9)
Associated arrhythmia surgery	2116 (2.9)	271 (9.3)	2387 (3.1)

Number and proportion (%) or mean and SD.

CABG, coronary artery bypass grafting; LVEF, left ventricular ejection fraction.

### Incidence of PPM following cardiac surgery

The cumulative incidence of PPM implantation was 2.9% at 30 days (n=2196), 3.8% at 1 year (n=2903), and 9.5% at 10 years (n=5722) following cardiac surgery. The median time to PPM implantation was 10 days (IQR 7–34 days) for those who received a PPM during the first postoperative year and 8 days for those who received a PPM within 30 days. Baseline characteristics for patients with and without PPM at 1 year from cardiac surgery are presented in table 1. Patients who received a PPM during the first year were generally older, more often female, had a lower left ventricular ejection fraction and had other surgery than isolated CABG compared with patients who did not get a PPM within the first year. The most common lead configuration implanted within 30 days was a dual-chamber pacemaker (85.4%, n=1875), a single-chamber pacemaker was implanted in 9.6% (n=211) of patients and 5.0% (n=110) of patients received a coronary sinus lead.

**Figure 2:**
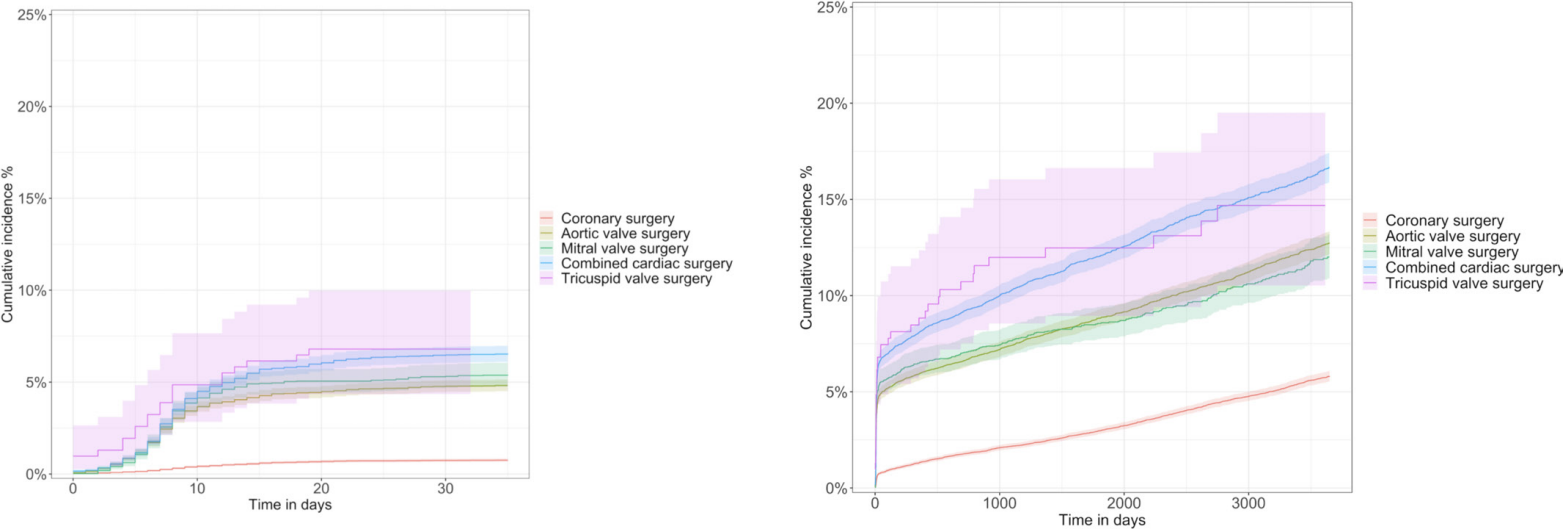
Cumulative incidence of permanent pacemaker implantation following cardiac surgery.

**Figure 3:**
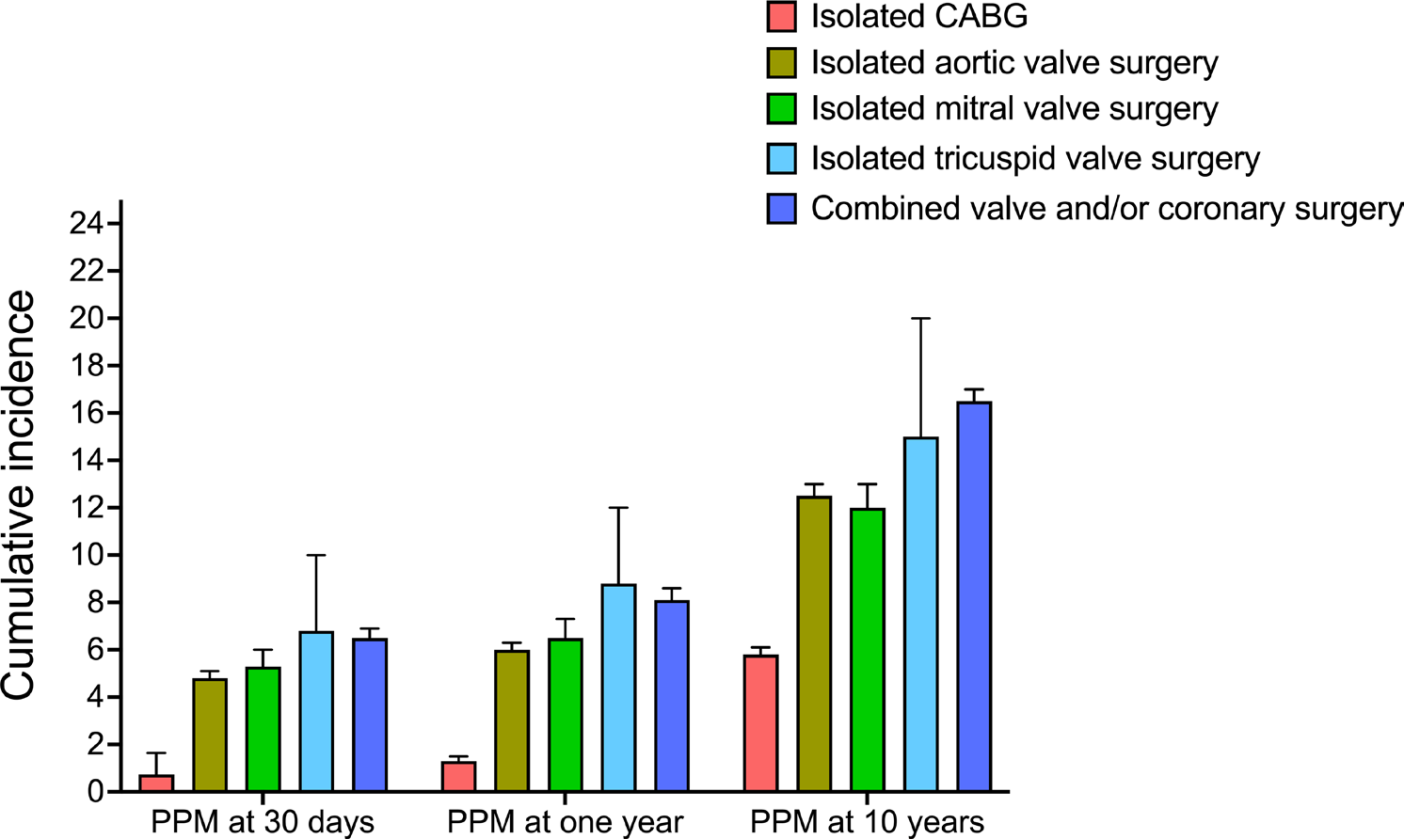
The cumulative incidence (with 95% CI) of PPM implantation at 30 days, 1 year and 10 years by type of cardiac surgery. CABG, coronary artery bypass grafting; PPM, permanent pacemaker.

**Figure 4:**
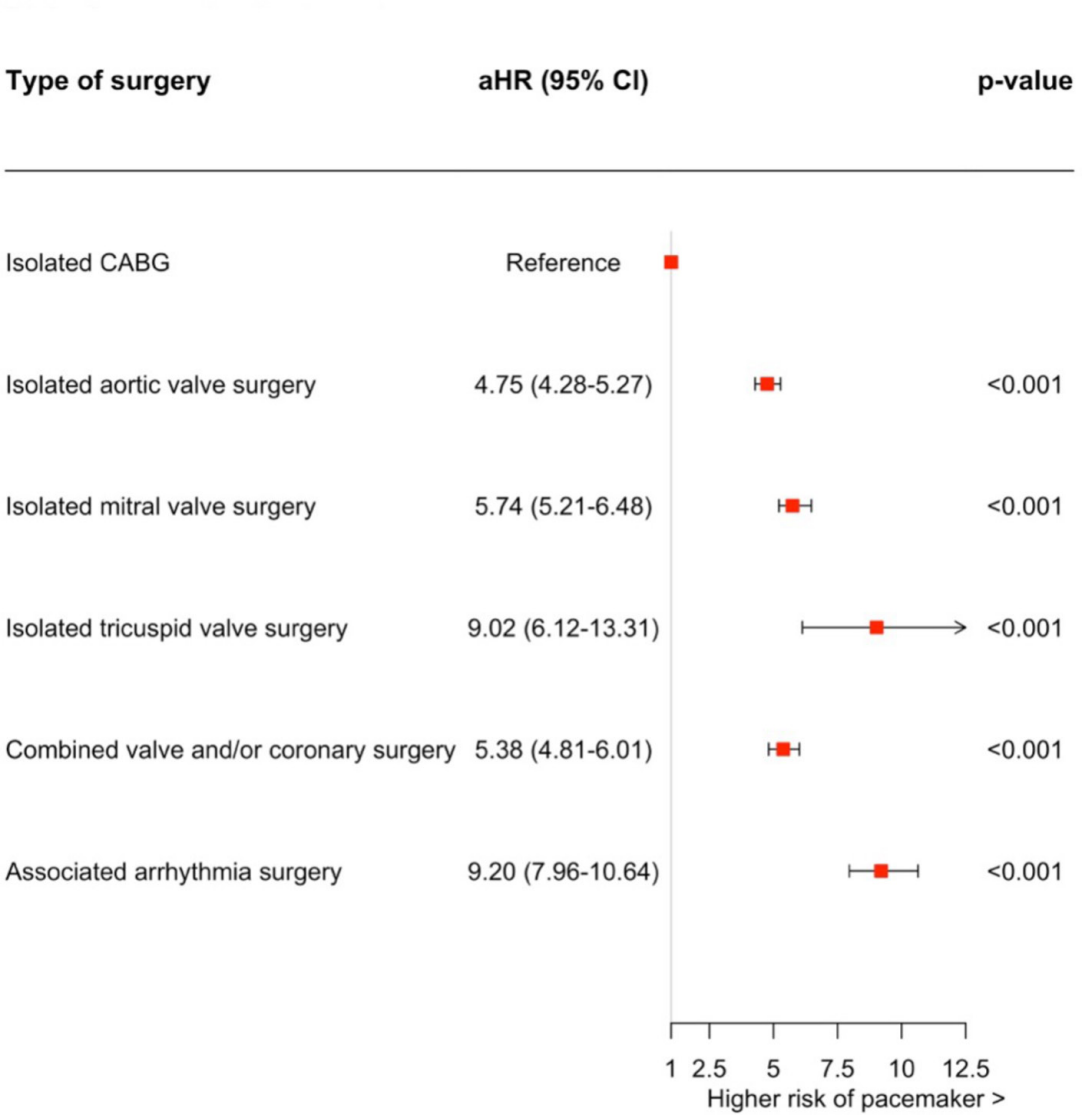
Forest plot illustrating the risk for permanent pacemaker, adjusted for age and sex, after different cardiac procedures with coronary artery bypass grafting as reference. aHR, adjusted HR; CABG, coronary artery bypass grafting.

Among isolated procedures, tricuspid valve surgery had the highest cumulative incidence of PPM implantation at both 30 days (6.8%, 95% CI 4.3% to 10.0%) and 1 year (8.8%, 95% CI 6.0% to 12.0%). The cumulative incidence of PPM implantation was 5.3% (95% CI 4.7% to 6.0%) at 30 days and 6.5% (95% CI 5.8% to 7.3%) at 1 year following isolated mitral valve surgery, 4.8% (95% CI 4.5% to 5.1%) at 30 days and 6.0% (95% CI 5.6% to 6.3%) at 1 year after aortic valve surgery, and 0.74% (95% CI 0.6% to 0.8%) at 30 days and 1.3% (95% CI 1.2% to 1.3%) at 1 year following CABG (figure 2). In patients who underwent a combined valve and/or coronary surgery, the incidence of PPM was 6.5% (95% CI 6.0% to 6.9%) and 8.1% (95% CI 7.7% to 8.6%) at 30 days and 1 year, respectively. The prevalence of PPM increased over time in all cardiac surgery groups (figure 3). The incidence of PPM implantation was highest when ablation surgery was performed alongside other procedures, significantly increasing the risk of PPM implantation across all procedure combinations. A detailed description of the cumulative incidence of PPM implantation following all the different combinations of cardiac surgical procedures is presented in online supplemental table S1. Figure 4 presents 1 year PPM implantation estimates, adjusted for age and sex, with isolated CABG as the reference. All other procedures showed a significantly higher 1 year PPM risk, with arrhythmia surgery having the highest (aHR 9.20, 95% CI 7.96 to 10.64, p<0.001). When evaluating the addition of arrhythmia surgery to the different types of other surgery groups, an increased risk of future PPM implantation was noted for all surgery groups except for isolated tricuspid valve surgery, which had similar point estimates (table 2).

**Table 2:** Associations between the addition of arrhythmia surgery and risk of pacemaker within 1 year

Type of surgery	aHR (95% CI)	P value
Isolated CABG	2.44 (1.46 to 4.08)	<0.001
Isolated aortic valve surgery	1.87 (1.44 to 2.43)	<0.001
Isolated mitral valve surgery	1.54 (1.18 to 1.99)	0.001
Isolated tricuspid valve surgery	2.00 (0.92 to 4.33)	0.080
Combined cardiac surgery	2.55 (2.10 to 3.11)	<0.001
Any surgery	3.30 (2.91 to 3.74)	<0.001

Cox regression analysis adjusted for age and sex.

aHR, adjusted HR; CABG, coronary artery bypass grafting.

The 30-day cumulative incidence of PPM implantation for patients per calendar year is depicted in online supplemental figure S1 and table S2. There was an increase in overall pacemaker implantation over the years (p<0.001). When stratified based on the surgery group, the change was less apparent due to greater variability caused by a lower number of patients in each subgroup (online supplemental figure S2).

In a cumulative risk regression analysis, stratified for each surgery group and adjusted for age and sex, operation in later years was associated with a higher incidence of PPM in patients with combined valve or valve and CABG (HR 1.02, 95% CI 1.00 to 1.03 per year increase, p=0.016) and aortic valve surgery (HR 1.02, 95% CI 1.00 to 1.03 per year increase, p<0.001) but not for isolated CABG (HR 1.00, 95% CI 0.99 to 1.01 per year increase, p=0.78), concomitant surgical ablation (HR 1.01, 95% CI 0.98 to 1.03 per year increase, p=0.69), mitral valve surgery (HR 1.00, 95% CI 0.98 to 1.02) per year increase, p=0.81) or tricuspid valve surgery (HR 0.95, 95% CI 0.88 to 1.02).

The risk for PPM implantation beyond the first postoperative year for patients who survived this period (n=66 581) was compared with age-matched and sex-matched controls (n=66 581) with no previous cardiac surgery. The aHR for the risk of PPM implantation in patients with previous cardiac surgery was 3.75 (95% CI 3.50 to 4.01, online supplemental table S3).

### Indication for PPM implantation

Atrioventricular block was the main indication in 65.1% of the patients in whom a PPM was implanted within the first postoperative year. Following isolated tricuspid valve, isolated aortic valve, isolated mitral valve and combined valve and coronary surgery, atrioventricular block was the predominant indication. Sinus node dysfunction was the main indication when surgical ablation was performed, whereas following CABG, the distribution of these two indications was similar (table 3). Similar trends were found for patients who received a PPM within 30 days.

**Table 3:** Indications for pacemaker implantation by the type of cardiac surgery

Indication	Total (N=2903)	Isolated CABG (n=554)	Isolated aortic valve surgery (n=1025)	Isolated mitral valve surgery (n=304)	Isolated tricuspid valve surgery (n=27)	Combined valve and/or coronary surgery (n=722)	Associated arrhythmia surgery (n=271)
30 days							
AVB	1497 (68.2%)	143 (46.1%)	665 (80.8%)	152 (61.0%)	19 (90.5%)	402 (69.9%)	116 (53.2%)
SND	699 (31.8%)	167 (53.9%)	158 (19.2%)	97 (39.0%)	2 (9.5%)	173 (30.1%%)	102 (46.8%)
1 year							
AVB	1891 (65.1%)	277 (50.0%)	786 (76.7%)	180 (59.2%)	23 (85.2%)	495 (68.6%)	130 (48.0%)
SND	1012 (34.9%)	277 (50.0%)	239 (23.3%)	124 (40.8%)	4 (14.8%)	227 (31.4%)	141 (52.0%)

AVB, atrioventricular block; CABG, coronary artery bypass grafting; SND, sinus node disease.

## Discussion

The main findings of this comprehensive, nationwide study are (1) the overall risk for PPM implantation within 1 year after cardiac surgery is 3.8%, mostly due to atrioventricular block, with incidence variation depending on the type of procedure performed. (2) Among isolated procedures, tricuspid valve surgery is associated with the highest risk of PPM, while combined valve, valve plus CABG and concomitant surgical ablation are associated with the highest risk.

Previous research on PPM implantation rates after cardiac surgery, mainly from outdated cohorts, shows considerable variability^3 5^ due to selective surgery inclusion,^4 9^ short follow-up periods^17^ or inclusion of various cardiac devices.^7^ This study provides comprehensive, contemporary data on PPM implantation rates and indications across cardiac surgeries with complete follow-up. The findings offer valuable insights for physicians when discussing PPM risks with patients and highlight procedures where prolonged rhythm monitoring might be needed.

Among patients who underwent isolated procedures, tricuspid valve surgery was associated with the highest incidence of PPM implantation. This might be explained by the proximity of the tricuspid valve to the atrioventricular node and bundle of HIS, which could be affected by the surgical trauma leading to postoperative bradyarrhythmias. However, isolated tricuspid valve surgery accounted for only a small fraction of cases (0.4%), and data for these patients should be interpreted cautiously. In this study, aortic valve surgery was the most frequently performed valvular procedure, with a 6.0% incidence of PPM implantation at 1 year. The combination of its high procedural volume and comparatively elevated PPM implantation rate underscores its clinical significance. Randomised controlled trials on transcatheter aortic valve replacement (TAVR), including the PARTNER 3 trial^18^ and the EVOLUT trial^19^ reported similar PPM implantation rates as the current study. Although the PARTNER 3 trial showed no significant difference in the need for new PPM implantation between patients who underwent surgical aortic valve replacement and those who underwent TAVR, other studies^20^ showed that TAVR was associated with a higher risk of PPM implantation. Given the increasing burden of valvular heart disease,^21^ the short-term and long-term PPM-related complications,^22^ and the presence of pre-existing conduction disturbances in an ageing population undergoing more aortic valve interventions, strategies to minimise intraprocedural conduction damage are essential. Another finding of note in our study was that patients who underwent concomitant surgical ablation had approximately nine times higher risk for PPM implantation, with sinus node dysfunction being the main indication in this group, in line with previous studies.^17 23^ This association was notable in all types of surgery except for isolated tricuspid valve surgery. Concomitant surgical atrial fibrillation (AF) ablation in patients undergoing valve surgery is recommended by international guidelines.^24 25^ Additionally, the European Heart Rhythm Association’s new consensus statement strongly recommends surgical ablation for paroxysmal or persistent AF during left atrial surgery.^26^ This document, however, based the perceived risk of PPM implantation associated with surgical AF ablation primarily on a study where only 295 (2.5%) out of 11 949 patients required a PPM after cardiac surgery.^27^ Studies comparing surgical and catheter ablation have, however, shown mixed results. The FAST trial (Atrial Fibrillation Catheter Ablation vs Surgical Ablation Treatment) randomised 124 patients to either stand-alone surgical or catheter AF ablation.^28^ Surgical ablation was superior to catheter ablation in achieving freedom from AF at 12 months but associated with higher complication rates, mainly driven by major bleedings and periprocedural pacemakers. Later PPM implantation rates were not reported. A recent randomised trial found catheter and surgical ablation similarly effective, with no adverse events in the catheter group compared with 20.8% in the surgical group.^29^ Potential complications, including the risk for PPM implantation, should, therefore, be considered before surgical AF ablation. However, it should be noted that patients undergoing arrhythmia surgery likely represent a unique group with a higher inherent risk of bradyarrhythmias.

The incidence of PPM implantation increased over the study period, indicating that pacemaker implantations after cardiac surgery are unlikely to decrease over the coming years. This observed trend is likely driven by the ageing population and the growing number of cardiac surgeries in elderly, frail patients.^30^

Finally, this risk for PPM implantation after the first postoperative year was more than threefold higher compared with an age-matched and sex-matched cohort without a history of cardiac surgery. The intention of this analysis was merely to confirm that the risk for PPM implantation following cardiac surgery remains markedly elevated even beyond the first postoperative year. The current analyses cannot estimate whether the increased HRs are caused by the residual risk associated with cardiac surgery or by the prevalence of concomitant heart disease and comorbidity in the surgery group. During follow-up, careful monitoring should, nevertheless, be considered to detect bradyarrhythmias.

### Limitations and strengths

Data on pre-existing conduction abnormalities, such as bundle branch block, were not available. Furthermore, information on the use of certain medications (eg, beta-blockers or antiarrhythmic drugs), which could influence the risk for PPM implantation, was lacking. Additionally, information regarding pacemaker stimulation rates and the potential recovery of conduction disturbances following PPM implantation was unavailable. This study, however, outlines real-world rates of PPM implantation after cardiac surgery across multiple centres in a large contemporary nationwide cohort using data from high-quality mandatory registries.

## Conclusions

The need for PPM after cardiac surgery is common and mostly due to atrioventricular block. Among isolated cardiac procedures, tricuspid valve surgery is associated with the highest risk for PPM. Both combined cardiac procedures and concomitant surgical ablation further increase that risk.

## Supplementary material

10.1136/heartjnl-2024-325321 online supplemental file 1

## Data Availability

Data are available upon reasonable request.
