# Supplementary material for: Pacemaker implantation after cardiac surgery: a contemporary, nationwide perspective
Source: Heart. 2025 Mar 29;111(21):e325321. doi: 10.1136/heartjnl-2024-325321 (PMC12573347; doi:10.1136/heartjnl-2024-325321)

**SUPPLEMENTARY MATERIAL**

**Table S1. The cumulative incidence with 95% confidence interval of permanent pacemaker implantation at 30 days, one-year and 10 years according to the type of cardiac surgery.**

| **Type of cardiac surgery** | **Days 30** | **Days 365** | **Days 3650** |
| --- | --- | --- | --- |
| Aortic valve and arrhythmia surgery | 9.5% (7.2%, 12%) | 11% (8.5%, 14%) | 20% (16%, 24%) |
| Aortic valve and mitral valve surgery | 11% (8.6%, 13%) | 13% (10%, 16%) | 23% (19%, 27%) |
| Aortic valve and tricuspid valve surgery | 14% (8.2%, 22%) | 16% (9.8%, 24%) | 25% (15%, 36%) |
| Aortic valve, mitral valve and arrhythmia surgery | 5.3% (1.4%, 13%) | 11% (4.3%, 20%) | 29% (17%, 43%) |
| Aortic valve, mitral valve and tricuspid valve surgery | 15% (7.5%, 24%) | 18% (9.7%, 28%) | 24% (14%, 36%) |
| Aortic valve, mitral valve, tricuspid valve and arrhythmia surgery | 19% (4.3%, 41%) | 19% (4.3%, 41%) | 26% (7.5%, 50%) |
| Aortic valve, tricuspid valve and arrhythmia surgery | 21% (6.2%, 42%) | 26% (9.2%, 47%) | 33% (13%, 54%) |
| CABG and aortic valve surgery | 4.1% (3.6%, 4.6%) | 5.4% (4.9%, 6.0%) | 14% (13%, 15%) |
| CABG and arrhythmia surgery | 2.2% (1.1%, 3.9%) | 3.3% (1.9%, 5.3%) | 13% (9.7%, 17%) |
| CABG and mitral valve surgery | 5.7% (4.4%, 7.3%) | 7.6% (6.0%, 9.3%) | 14% (12%, 16%) |
| CABG and tricuspid valve surgery | 4.8% (0.84%, 14%) | 4.8% (0.84%, 14%) | 13% (3.4%, 30%) |
| CABG, aortic and mitral valve surgery | 13% (8.1%, 20%) | 13% (8.1%, 20%) | 23% (14%, 32%) |
| CABG, aortic valve and arrhythmia surgery | 6.7% (3.1%, 12%) | 9.3% (4.9%, 15%) | 18% (11%, 26%) |
| CABG, aortic valve and tricuspid valve surgery | 3.7% (0.25%, 16%) | 7.4% (1.2%, 21%) | 12% (2.8%, 28%) |
| CABG, aortic valve, mitral valve and arrhythmia surgery | 18% (2.5%, 46%) | 27% (5.8%, 55%) | 36% (10%, 64%) |
| CABG, aortic valve, mitral valve and tricuspid valve surgery | 20% (6.0%, 40%) | 20% (6.0%, 40%) | 20% (6.0%, 40%) |
| CABG, aortic valve, mitral valve, tricuspid valve and arrhythmia surgery | 0.00% (—%, —%) | 0.00% (—%, —%) | —% (—%, —%) |
| CABG, aortic valve, tricuspid valve, and arrhythmia surgery | 0.00% (—%, —%) | 0.00% (—%, —%) | —% (—%, —%) |
| CABG, mitral valve and arrhythmia surgery | 12% (6.9%, 19%) | 15% (9.0%, 23%) | 30% (20%, 39%) |
| CABG, mitral valve and tricuspid valve surgery | 14% (8.8%, 21%) | 17% (11%, 25%) | 20% (13%, 29%) |
| CABG, mitral valve, tricuspid valve, and arrhythmia surgery | 17% (6.1%, 33%) | 17% (6.1%, 33%) | 26% (11%, 45%) |
| CABG, tricuspid valve, and arrhythmia surgery | 40% (3.1%, 79%) | 40% (3.1%, 79%) | —% (—%, —%) |
| Isolated aortic valve surgery | 4.8% (4.5%, 5.1%) | 6.0% (5.7%, 6.4%) | 12% (12%, 13%) |
| Isolated CABG | 0.73% (0.65%, 0.82%) | 1.3% (1.2%, 1.5%) | 5.9% (5.6%, 6.2%) |
| Isolated mitral valve surgery | 5.2% (4.6%, 5.9%) | 6.3% (5.6%, 7.1%) | 12% (11%, 13%) |
| Isolated tricuspid valve surgery | 7.0% (4.4%, 10%) | 9.2% (6.2%, 13%) | 14% (9.9%, 19%) |
| Mitral valve and arrhythmia surgery | 7.3% (5.6%, 9.4%) | 10% (8.1%, 13%) | 20% (17%, 24%) |
| Mitral valve and tricuspid surgery | 16% (13%, 19%) | 18% (15%, 21%) | 23% (20%, 28%) |
| Mitral valve, tricuspid valve and arrhythmia surgery | 21% (16%, 26%) | 24% (19%, 29%) | 35% (28%, 41%) |
| Tricuspid valve and arrhythmia surgery | 19% (9.2%, 31%) | 19% (9.2%, 31%) | 34% (20%, 49%) |

**Table S2.** **Cumulative incidence in different years, stratified per surgery group.**

| **Characteristic** | **Days 30** | **Days 365** | **Days 3650** |
| --- | --- | --- | --- |
| Year, type of surgery |  |  |  |
| 2006, Associated arrhythmia surgery | 3.0% (0.22%, 14%) | 9.1% (2.3%, 22%) | 18% (7.2%, 33%) |
| 2007, Associated arrhythmia surgery | 9.0% (5.5%, 13%) | 12% (8.3%, 17%) | 20% (15%, 26%) |
| 2008, Associated arrhythmia surgery | 6.6% (3.8%, 10%) | 8.3% (5.2%, 12%) | 17% (12%, 22%) |
| 2009, Associated arrhythmia surgery | 8.3% (5.2%, 12%) | 11% (7.7%, 16%) | 22% (17%, 27%) |
| 2010, Associated arrhythmia surgery | 11% (6.9%, 16%) | 13% (8.3%, 18%) | 25% (19%, 32%) |
| 2011, Associated arrhythmia surgery | 10% (6.1%, 16%) | 13% (8.1%, 19%) | —% (—%, —%) |
| 2012, Associated arrhythmia surgery | 13% (8.3%, 19%) | 16% (11%, 22%) | —% (—%, —%) |
| 2013, Associated arrhythmia surgery | 12% (7.5%, 18%) | 12% (7.5%, 18%) | —% (—%, —%) |
| 2014, Associated arrhythmia surgery | 7.9% (4.5%, 12%) | 12% (7.6%, 17%) | —% (—%, —%) |
| 2015, Associated arrhythmia surgery | 7.8% (4.4%, 12%) | 10% (6.2%, 15%) | —% (—%, —%) |
| 2016, Associated arrhythmia surgery | 7.0% (3.6%, 12%) | 9.1% (5.1%, 15%) | —% (—%, —%) |
| 2017, Associated arrhythmia surgery | 8.5% (4.9%, 13%) | 9.7% (5.8%, 15%) | —% (—%, —%) |
| 2018, Associated arrhythmia surgery | 11% (6.7%, 16%) | 12% (7.7%, 18%) | —% (—%, —%) |
| 2019, Associated arrhythmia surgery | 9.4% (5.2%, 15%) | 12% (6.9%, 17%) | —% (—%, —%) |
| 2020, Associated arrhythmia surgery | 9.2% (4.7%, 16%) | —% (—%, —%) | —% (—%, —%) |
| 2006, Combined cardiac surgery | 4.2% (2.9%, 5.7%) | 5.5% (4.1%, 7.3%) | 15% (13%, 18%) |
| 2007, Combined cardiac surgery | 4.9% (3.3%, 6.9%) | 5.8% (4.0%, 7.9%) | 12% (9.4%, 15%) |
| 2008, Combined cardiac surgery | 5.7% (4.0%, 7.7%) | 7.0% (5.2%, 9.2%) | 13% (10%, 15%) |
| 2009, Combined cardiac surgery | 6.6% (5.0%, 8.6%) | 7.9% (6.1%, 10%) | 16% (13%, 19%) |
| 2010, Combined cardiac surgery | 4.7% (3.3%, 6.3%) | 7.4% (5.7%, 9.4%) | 14% (12%, 17%) |
| 2011, Combined cardiac surgery | 4.8% (3.4%, 6.6%) | 6.1% (4.5%, 8.1%) | 14% (11%, 17%) |
| 2012, Combined cardiac surgery | 6.0% (4.4%, 7.9%) | 7.3% (5.5%, 9.4%) | —% (—%, —%) |
| 2013, Combined cardiac surgery | 6.0% (4.4%, 8.0%) | 7.5% (5.7%, 9.6%) | —% (—%, —%) |
| 2014, Combined cardiac surgery | 6.0% (4.3%, 8.0%) | 7.5% (5.7%, 9.8%) | —% (—%, —%) |
| 2015, Combined cardiac surgery | 5.7% (4.1%, 7.6%) | 7.0% (5.3%, 9.1%) | —% (—%, —%) |
| 2016, Combined cardiac surgery | 5.9% (4.2%, 7.9%) | 7.3% (5.4%, 9.6%) | —% (—%, —%) |
| 2017, Combined cardiac surgery | 6.8% (5.1%, 8.8%) | 8.4% (6.5%, 11%) | —% (—%, —%) |
| 2018, Combined cardiac surgery | 6.7% (4.9%, 8.8%) | 7.7% (5.7%, 9.9%) | —% (—%, —%) |
| 2019, Combined cardiac surgery | 6.3% (4.5%, 8.4%) | 8.1% (6.1%, 11%) | —% (—%, —%) |
| 2020, Combined cardiac surgery | 8.2% (6.0%, 11%) | —% (—%, —%) | —% (—%, —%) |
| 2006, Isolated aortic valve surgery | 4.3% (3.2%, 5.7%) | 5.3% (4.0%, 6.8%) | 12% (9.6%, 14%) |
| 2007, Isolated aortic valve surgery | 4.3% (3.2%, 5.5%) | 5.4% (4.2%, 6.8%) | 11% (9.4%, 13%) |
| 2008, Isolated aortic valve surgery | 4.3% (3.2%, 5.6%) | 5.1% (3.9%, 6.6%) | 11% (9.0%, 13%) |
| 2009, Isolated aortic valve surgery | 4.0% (2.9%, 5.3%) | 5.7% (4.5%, 7.3%) | 11% (9.3%, 13%) |
| 2010, Isolated aortic valve surgery | 4.6% (3.5%, 5.9%) | 5.9% (4.6%, 7.4%) | 11% (9.4%, 13%) |
| 2011, Isolated aortic valve surgery | 4.1% (3.1%, 5.3%) | 5.2% (4.0%, 6.5%) | 16% (11%, 22%) |
| 2012, Isolated aortic valve surgery | 4.3% (3.3%, 5.5%) | 5.4% (4.2%, 6.7%) | —% (—%, —%) |
| 2013, Isolated aortic valve surgery | 4.7% (3.6%, 6.0%) | 6.0% (4.7%, 7.5%) | —% (—%, —%) |
| 2014, Isolated aortic valve surgery | 3.8% (2.8%, 5.0%) | 5.2% (4.0%, 6.5%) | —% (—%, —%) |
| 2015, Isolated aortic valve surgery | 7.2% (5.8%, 8.8%) | 8.2% (6.7%, 9.8%) | —% (—%, —%) |
| 2016, Isolated aortic valve surgery | 4.2% (3.1%, 5.4%) | 5.6% (4.4%, 7.0%) | —% (—%, —%) |
| 2017, Isolated aortic valve surgery | 5.7% (4.5%, 7.2%) | 6.9% (5.6%, 8.5%) | —% (—%, —%) |
| 2018, Isolated aortic valve surgery | 5.7% (4.5%, 7.1%) | 6.6% (5.3%, 8.0%) | —% (—%, —%) |
| 2019, Isolated aortic valve surgery | 5.3% (4.1%, 6.7%) | 6.5% (5.2%, 8.1%) | —% (—%, —%) |
| 2020, Isolated aortic valve surgery | 5.0% (3.8%, 6.5%) | 7.3% (5.4%, 9.7%) | —% (—%, —%) |
| 2006, Isolated CABG | 0.80% (0.56%, 1.1%) | 1.3% (0.96%, 1.7%) | 5.3% (4.6%, 6.0%) |
| 2007, Isolated CABG | 0.44% (0.27%, 0.69%) | 1.1% (0.77%, 1.4%) | 5.4% (4.7%, 6.2%) |
| 2008, Isolated CABG | 0.40% (0.23%, 0.65%) | 1.1% (0.82%, 1.5%) | 5.5% (4.8%, 6.3%) |
| 2009, Isolated CABG | 0.58% (0.36%, 0.89%) | 1.1% (0.79%, 1.5%) | 5.0% (4.3%, 5.8%) |
| 2010, Isolated CABG | 0.85% (0.57%, 1.2%) | 1.5% (1.1%, 2.0%) | 5.8% (5.0%, 6.7%) |
| 2011, Isolated CABG | 0.93% (0.62%, 1.3%) | 1.4% (0.98%, 1.8%) | 7.9% (5.7%, 11%) |
| 2012, Isolated CABG | 0.57% (0.34%, 0.92%) | 1.1% (0.76%, 1.6%) | —% (—%, —%) |
| 2013, Isolated CABG | 0.56% (0.32%, 0.91%) | 0.95% (0.63%, 1.4%) | —% (—%, —%) |
| 2014, Isolated CABG | 0.93% (0.60%, 1.4%) | 1.5% (1.1%, 2.1%) | —% (—%, —%) |
| 2015, Isolated CABG | 0.92% (0.60%, 1.4%) | 1.6% (1.2%, 2.1%) | —% (—%, —%) |
| 2016, Isolated CABG | 0.81% (0.51%, 1.2%) | 1.5% (1.1%, 2.1%) | —% (—%, —%) |
| 2017, Isolated CABG | 0.99% (0.65%, 1.4%) | 1.8% (1.3%, 2.4%) | —% (—%, —%) |
| 2018, Isolated CABG | 0.83% (0.52%, 1.3%) | 1.3% (0.89%, 1.8%) | —% (—%, —%) |
| 2019, Isolated CABG | 0.97% (0.64%, 1.4%) | 1.5% (1.1%, 2.0%) | —% (—%, —%) |
| 2020, Isolated CABG | 0.88% (0.54%, 1.4%) | 3.6% (1.6%, 7.1%) | —% (—%, —%) |
| 2006, Isolated mitral valve surgery | 6.8% (4.3%, 9.9%) | 9.4% (6.4%, 13%) | 15% (11%, 19%) |
| 2007, Isolated mitral valve surgery | 4.8% (2.9%, 7.5%) | 6.7% (4.3%, 9.7%) | 12% (9.2%, 16%) |
| 2008, Isolated mitral valve surgery | 3.4% (1.7%, 6.1%) | 4.1% (2.2%, 7.0%) | 9.0% (6.0%, 13%) |
| 2009, Isolated mitral valve surgery | 6.1% (3.6%, 9.5%) | 6.5% (3.9%, 9.9%) | 12% (8.0%, 16%) |
| 2010, Isolated mitral valve surgery | 5.2% (3.1%, 8.2%) | 6.3% (3.9%, 9.5%) | 11% (7.8%, 15%) |
| 2011, Isolated mitral valve surgery | 2.4% (1.0%, 4.9%) | 4.4% (2.3%, 7.5%) | —% (—%, —%) |
| 2012, Isolated mitral valve surgery | 4.9% (2.8%, 7.8%) | 7.0% (4.4%, 10%) | —% (—%, —%) |
| 2013, Isolated mitral valve surgery | 6.0% (3.6%, 9.3%) | 6.8% (4.2%, 10%) | —% (—%, —%) |
| 2014, Isolated mitral valve surgery | 4.0% (2.1%, 6.8%) | 4.7% (2.6%, 7.7%) | —% (—%, —%) |
| 2015, Isolated mitral valve surgery | 6.0% (3.6%, 9.2%) | 6.7% (4.2%, 10%) | —% (—%, —%) |
| 2016, Isolated mitral valve surgery | 4.6% (2.7%, 7.1%) | 4.8% (2.9%, 7.4%) | —% (—%, —%) |
| 2017, Isolated mitral valve surgery | 4.2% (2.4%, 6.8%) | 5.5% (3.4%, 8.3%) | —% (—%, —%) |
| 2018, Isolated mitral valve surgery | 7.0% (4.7%, 10%) | 7.3% (5.0%, 10%) | —% (—%, —%) |
| 2019, Isolated mitral valve surgery | 6.3% (4.2%, 8.9%) | 8.2% (5.8%, 11%) | —% (—%, —%) |
| 2020, Isolated mitral valve surgery | 6.4% (4.3%, 9.0%) | 8.0% (5.4%, 11%) | —% (—%, —%) |
| 2006, Isolated tricuspid valve surgery | 4.5% (0.29%, 19%) | 4.5% (0.29%, 19%) | 18% (5.5%, 37%) |
| 2007, Isolated tricuspid valve surgery | 5.9% (0.35%, 24%) | 18% (4.1%, 39%) | 24% (6.9%, 46%) |
| 2008, Isolated tricuspid valve surgery | 9.1% (0.44%, 35%) | 18% (2.5%, 46%) | 18% (2.5%, 46%) |
| 2009, Isolated tricuspid valve surgery | 14% (2.1%, 38%) | 14% (2.1%, 38%) | 21% (4.7%, 46%) |
| 2010, Isolated tricuspid valve surgery | 20% (4.5%, 43%) | 27% (7.7%, 51%) | 33% (11%, 57%) |
| 2011, Isolated tricuspid valve surgery | 0.00% (—%, —%) | 0.00% (—%, —%) | —% (—%, —%) |
| 2012, Isolated tricuspid valve surgery | 0.00% (—%, —%) | 0.00% (—%, —%) | —% (—%, —%) |
| 2013, Isolated tricuspid valve surgery | 7.4% (1.2%, 21%) | 7.4% (1.2%, 21%) | —% (—%, —%) |
| 2014, Isolated tricuspid valve surgery | 0.00% (—%, —%) | 0.00% (—%, —%) | —% (—%, —%) |
| 2015, Isolated tricuspid valve surgery | 8.0% (1.3%, 23%) | 8.0% (1.3%, 23%) | —% (—%, —%) |
| 2016, Isolated tricuspid valve surgery | 6.3% (0.36%, 25%) | 6.3% (0.36%, 25%) | —% (—%, —%) |
| 2017, Isolated tricuspid valve surgery | 7.7% (1.3%, 22%) | 15% (4.7%, 32%) | —% (—%, —%) |
| 2018, Isolated tricuspid valve surgery | 10% (1.6%, 28%) | 10% (1.6%, 28%) | —% (—%, —%) |
| 2019, Isolated tricuspid valve surgery | 6.5% (1.1%, 19%) | 6.5% (1.1%, 19%) | —% (—%, —%) |
| 2020, Isolated tricuspid valve surgery | 6.7% (1.1%, 19%) | —% (—%, —%) | —% (—%, —%) |

**Table S3. Risk for permanent pacemaker in post-cardiac surgery patients (n=65,329) compared to controls (n=65,329) after the first postoperative year.**

|  | **HR (95% CI)** | **P-value** |
| --- | --- | --- |
| **Type of surgery** |  |  |
| Isolated CABG | 2.90 (2.68-3.13) | <0.001 |
| Isolated aortic valve surgery | 4.66 (4.26–5.10) | <0.001 |
| Isolated mitral valve surgery | 3.47 (3.32-4.08) | <0.001 |
| Isolated tricuspid valve surgery | 5.73 (3.32-9.90) | <0.001 |
| Combined valve and/or coronary surgery | 6.45 (5.83-7.13) | <0.001 |
| Associated arrhythmia surgery | 7.86 (6.72-9.20) | <0.001 |
| **Overall** | 3.75 (3.50-4.01) | <0.001 |

**Figure S1: 30-day cumulative incidence stratified by year of surgery.**

**
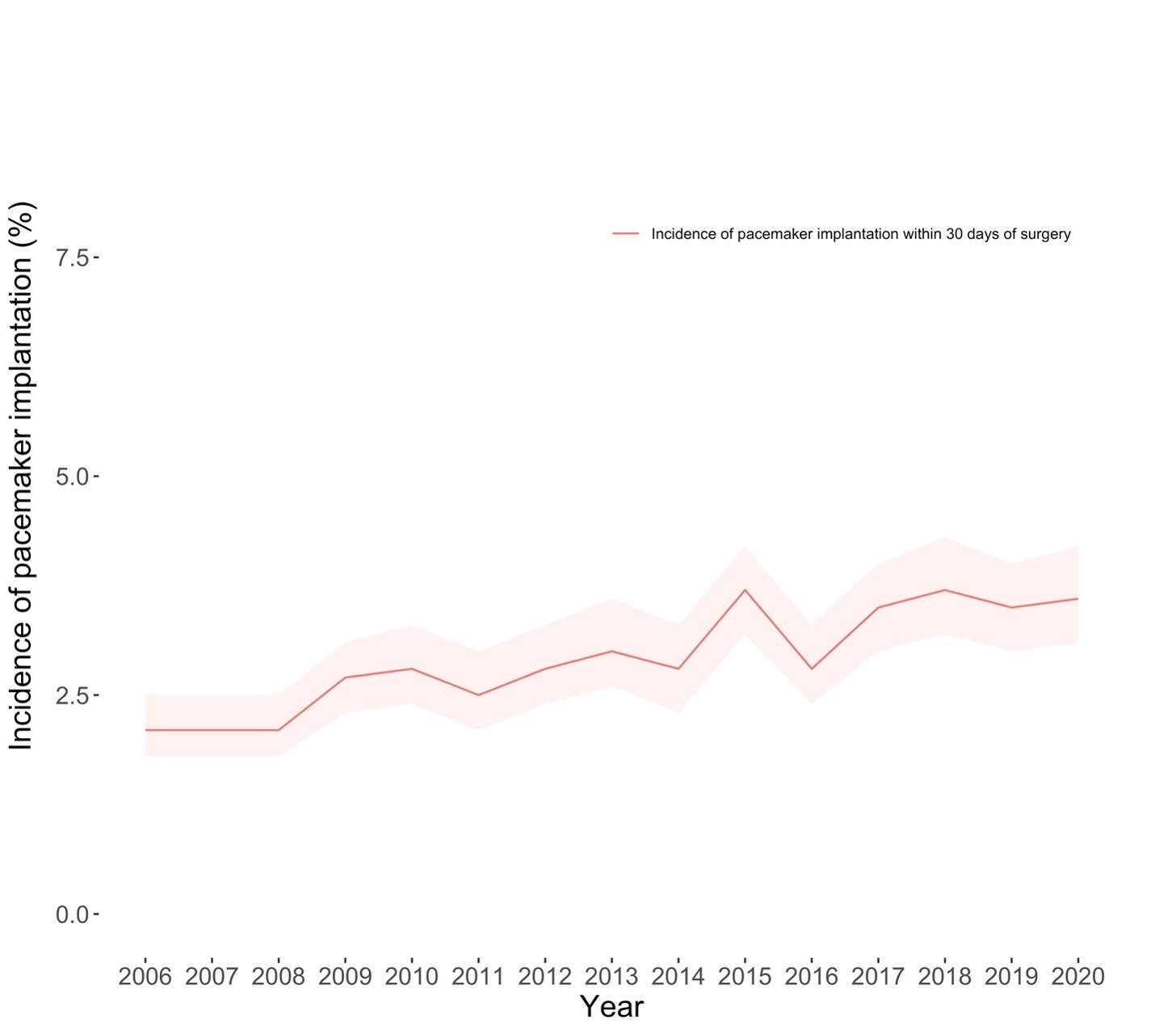
**

**Figure S2: 30-day cumulative incidence stratified on type and year of surgery (CABG: Coronary artery bypass grafting).**


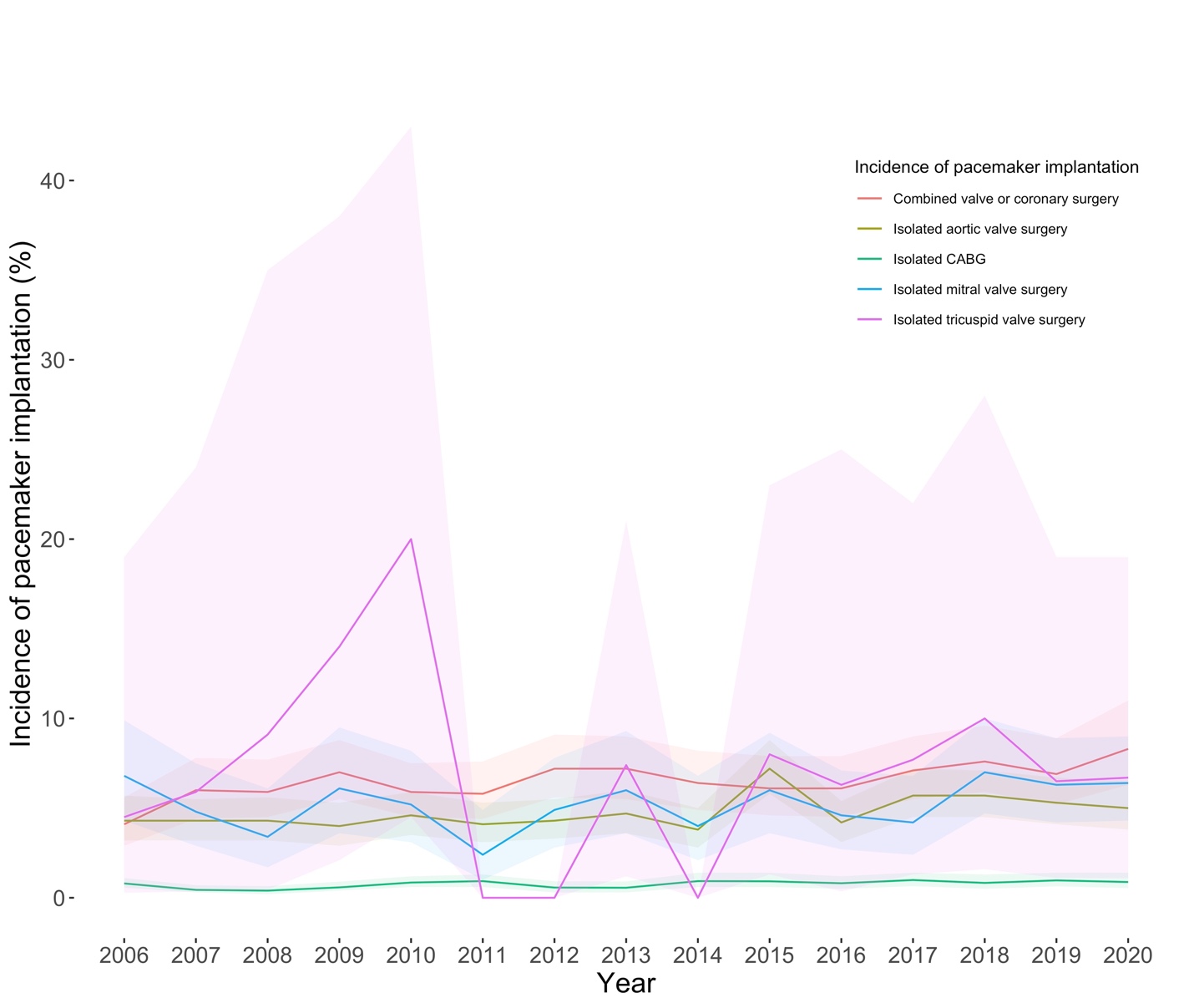

Supplement: online supplemental file 1 [file heartjnl-111-21-s001.docx]
